# Supplementary material for: Dissection of a rice OsMac1 mRNA 5’ UTR to uncover regulatory elements that are responsible for its efficient translation
Source: PLoS One. 2021 Jul 9;16(7):e0253488. doi: 10.1371/journal.pone.0253488 (PMC8270207; doi:10.1371/journal.pone.0253488)
Supplement: S1 Fig — (A) Nucleotide sequence of the control 5’ UTR, which is derived from pBI121 (AF485783). Numbers indicate the nucleotide position in the plasmid shown in the database. Regions for CaMV 35S promoter and the coding sequences of gusA gene are indicated by green and blue boxes, respectively. (B) Nucleotide sequences of the region around sp38. UTRc+31nt contains an additional 31 nt sequence derived from the 3’ portion of the intron 1. Gaps are introduced into the region lacking in each UTRs. sp38 in UTRc is indicated by an orange box. (PDF) [file pone.0253488.s001.pdf]

A

```
5701 GGATTGATGT GATATCTCCA CTGACGTAAG GGATGACGCA CAATCCCACT ATCCTTCGCA
5761 AGACCCTTCC TCTATATAAG GAAGTTCATT TCATTTGGAG AGAACACGGG GGACTCTAGA
5821 GGATCCCCGG GTGGTCAGTC CCTTATGTTA CGTCCTGTAG AAACCCCAAC CCGTGAAATC
5881 AAAAAACTCG ACGGCCTGTG GGCATTCAGT CTGGATCGCG AAAACTGTGG AATTGATCAG
```

B

```
UTRc+31nt  CUCCCUCAAG AAAC AAAATTTACC AGTTAAAAATG TTTCCAG CUACAAAA
UTRc       CUCCCUCAAG ---- ---------- ----------- ------- CUACAAAA
UTRb       CUCCCUCAAG ---- ---------- ----------- ------- --------
UTRa       CUCCCUCAAG ---- ---------- ----------- ------- --------

UTRc+31nt  AAAUACUCAG GUUUCAGAUC AUUUUUCGAG GUUGGAAUUG CUGCAG
UTRc       AAAUACUCAG GUUUCAGAUC AUUUUUCGAG GUUGGAAUUG CUGCAG
UTRb       ---------- ---------- ---------- GUUGGAAUUG CUGCAG
UTRa       ---------- ---------- ---------- ---------- ------

UTRc+31nt  GAUCAAGCGA AGCCUCGUCA GGCCAUGCCC UCGUCUCAU
UTRc       GAUCAAGCGA AGCCUCGUCA GGCCAUGCCC UCGUCUCAU
UTRb       GAUCAAGCGA AGCCUCGUCA GGCCAUGCCC UCGUCUCAU
UTRa       GAUCAAGCGA AGCCUCGUCA GGCCAUGCCC UCGUCUCAU
```

S1 Fig. (A) Nucleotide sequence of the control 5' UTR, which is derived from pBI121 (AF485783). Numbers indicate the nucleotide position in the plasmid shown in the database. Regions for CaMV 35S promoter and the coding sequence of *gusA* gene are indicated by green and blue boxes, respectively. (B) Nucleotide sequences of the region around sp38. UTRc+31nt contains an additional 31 nt sequence derived from the 3' portion of the intron 1. Gaps are introduced into the region lacking in each UTRs. sp38 in UTRc is indicated by an orange-colored box.
