# Supplementary material for: Dissection of a rice OsMac1 mRNA 5’ UTR to uncover regulatory elements that are responsible for its efficient translation
Source: PLoS One. 2021 Jul 9;16(7):e0253488. doi: 10.1371/journal.pone.0253488 (PMC8270207; doi:10.1371/journal.pone.0253488)
Supplement: S2 Fig — The CU-rich sequence in the 5’ region is indicated by blue letters. Nucleotide sequence of sp38 is shown in an orange box. Splicing sites in UTRa and UTRb are indicated by red triangles. uORFs are shown in red boxes. Initiation codons AUG are indicated by red letters. The downstream ORF of OsMac1 is shown in a yellow box. Nucleotide sequences consisting of Region–A and Regions–B are double-underlined. (PDF) [file pone.0253488.s002.pdf]

CU-rich region

1 ACGCUACGCC UACGCGGGAG GGUUCCUCCU CCUCCGCCCC CCAUCCAUCC AAAUCCAAUC 60

61 GCCGUCGUCG AUCCCCUCAG CCCUGCUGCC GCCGCCCCCU UUCAGCGCAA AUCGUCGAUU 120

121 UCCCCCGCCU CCAUCUCCCG CCGCUCGCCG CCGGGACCCC GCACCGCAAA UCGUCGUCCU 180

181 GCCUGCAUCU UCGAUUCCCU CCGCCGCCCG UGGUUCCGCU CCUUAGCUGC CCAUCUUCAC 240

A A

241 AUCUCCCUCA AG ====== Intron ======= CUA CAAAAAAAUA CUCAGGUUUC AGAUCAUUUU 285

B

sp38

286 UCGAGGUUGG AAUUGCUGCA GGAUCAAGCG AAGCCUCGUC AGGCCAUGCC CUCGUCUCAU 345

346 UCCUCAGCUU GCAGAGUCCA GUUCCAAGCA G ===== Intron ===== UUGAAUA CUACCGUGCA 393

394 UUUGGUGGAA GAACAUAAUC CUACCUCUUG GUGUCUGCCU CCUAA UAAAG GACUCCAGUA 453

454 UCAGUUGGGG CUUGUGAAUU AUGAUACUUU AUGAACCAUA UUGUGAAACA GGAGGAGGCU 513

A A

514 GUCAAUAUAU UGGUAGCUCU AAUAGUGGCG UACUUUGAGU AGAAUUGAGG GAGACAAGAG 573

A B

574 AGAACA AUGG AAUUGGCAGA GCAGCUGUAC UCAGUUUUCA GGAGCUAUGC GCCCCCUAUU 663

664 UGGGCUUCAA UCACCGCUGG CAUUUUUGUC AUCACCUCGC UUUCACUAUC UCUGUUCUUG 723

Downstream ORF (*OsMac1*)

uORF

S2 Fig. Nucleotide sequence of UTRc. The CU-rich sequence in the 5' region is indicated by blue-colored letters. Nucleotide sequence of sp38 is orange-colored. Splicing sites in UTRa and UTRb are indicated by red triangles. uORFs are boxed. Initiation codons AUG are indicated by red-colored letters. The downstream ORF of *OsMac1* is shown in a yellow-colored box. Nucleotide sequences consisting of Region–A and Regions–B are double-underlined.
