# Supplementary material for: Dissection of a rice OsMac1 mRNA 5’ UTR to uncover regulatory elements that are responsible for its efficient translation
Source: PLoS One. 2021 Jul 9;16(7):e0253488. doi: 10.1371/journal.pone.0253488 (PMC8270207; doi:10.1371/journal.pone.0253488)
Supplement: S3 Fig — mRNA for the reporters were obtained by RT-PCR immediately after testing their translation efficiencies. cDNA was amplified using primers corresponding the entire 5’UTR region and the GUS coding region. From left to right: cDNA (UTRc) contains the control DNA fragment amplified from the wild-type UTRc; UTRc indicates the transcript amplified from the wild-type UTRc construct; "Complementary", "Restored-interaction", and "Tuncation" shows corresponding mutant transcripts described in Fig 6. A Gap is introduced between the lanes of the same gel. Size markers used are 100 bp DNA ladder marker (New England Biolabs, Ipswich, MA, USA). (PDF) [file pone.0253488.s003.pdf]

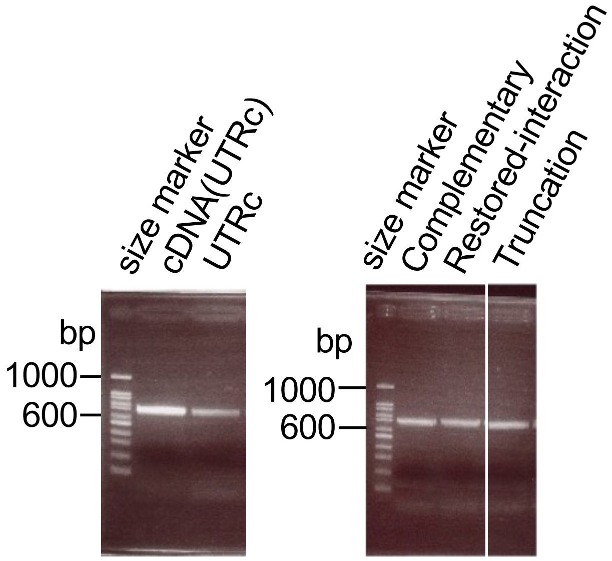

S3 Fig. Analysis of integrity of the reporter mRNA during rice suspension culture protoplast incubation. mRNA for the reporters were obtained by RT-PCR immediately after testing their translation efficiencies. cDNA was amplified using primers corresponding the entire 5'UTR region and the GUS coding region. From left to right: cDNA(UTRc) contains the control DNA fragment amplified from the wild-type UTRc; UTRc indicates the transcript amplified from the wild-type UTRc construct; "Complementary", "Restored-interaction", and "Tuncation" shows corresponding mutant transcripts described in Fig. 6. A gap is introduced between the lanes of the same gel. Size markers used are 100 bp DNA ladder marker (New England Biolabs, Ipswich, MA, USA).
