# Supplementary material for: Dissection of a rice OsMac1 mRNA 5’ UTR to uncover regulatory elements that are responsible for its efficient translation
Source: PLoS One. 2021 Jul 9;16(7):e0253488. doi: 10.1371/journal.pone.0253488 (PMC8270207; doi:10.1371/journal.pone.0253488)
Supplement: S1 Raw images — (PDF) [file pone.0253488.s004.pdf]

○ リバートラ

		×10
5×バッファー	8	80
dNTP (10mM)	4	40
Oligo	2	20
RevTra Ace	0.5	5
RNA	25.5	ごとMix.

14.5

total 40

全量 RNAが
うすくても 250～350くらい ng.
いつもは 500ng.

42	45:00
99	5:00
4	∞

一番左の古いPCR器.

23.6
1.2
472
236
283.2

↓

○ 半定量RT PCR.

cDNA	2
バッファー	2
dNTP	2
プライマー	2
B-Taq	0.2
milliQ	11.8
total 20.	

今回 cDNA量が少ないかもなので 2倍にした

いつもはコレ.
だけど…
今回はゲル切りして
シーケンスまでしたいから
40 μlでやる!

⇨

		×12	
cDNA	4	48	44
バッファー	4	48	✓44
dNTP (2mM)	4	48	44
プライマー	4	(24, 24)	✓22, 22
B-Taq	0.4	4.8	4.4
milliQ	23.6	283.2	✓259.6
	40.		

プライマー BamHI-UTRc-fw 64.7
(U19A230216)
GUS-D-70rev 52.0

P.C. に 35S::UTRc::GUS (8) を流そうかな.

1 P.C.
2 1 } UTRc
3 1'
4 2 } Mutation
5 2'
6 3 } No-interaction
7 3'
8 4 } Restored-interaction
9 4'
10 5 } Truncation
11 5'

1ng/μl

pDNA	2
バッファー	4
dNTP	4
プライマー	1/1 (2)
B-Taq	0.4
✓ milliQ	27.6
total 40 μl	

3.9
4.0
12.4
27.6

94	2:00	
94	0:30	28cycle
59	0:30	
72	0:30	
72	7:00	
8	∞	

上方から2コメ.

泳動 OK!

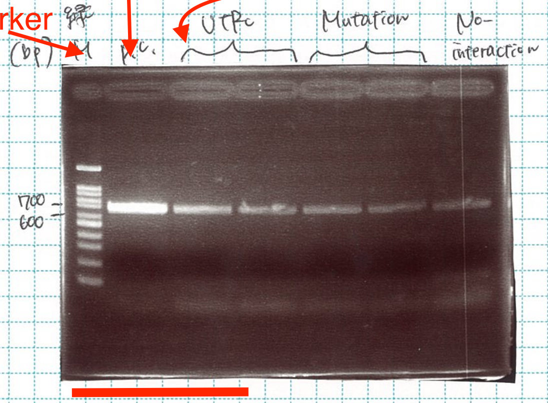

UTRc 580
Junctseq 24
GUS 70
674
+6 (BamHI)
680bp 増幅.

バンドサイズOK
&
バンドサイズに変化無いので、
スプライシングおきてないことを
証明できた。
truncationだけ (5'5(?))
ちょっと短いけど、言われてみると
そういうふうに見える.

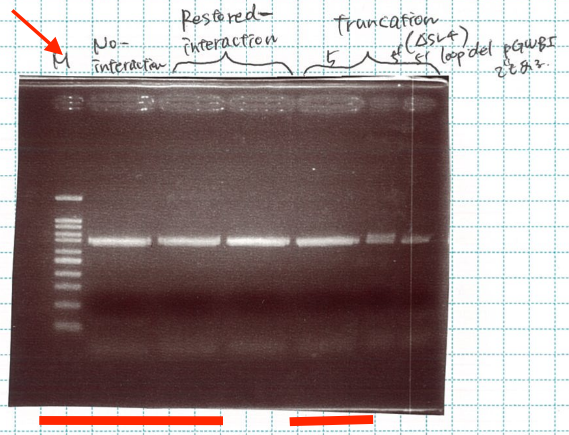

5'だけ2重に見えるっぽい
けど、5と元のRNAは全く同じ
だから問題無し!

先生に報告

Plant
に投稿だ～!と喜んでた。

pGWB5でもプロトプラスト形質転換できるんだなあ.
めちゃめちゃRNA少なくても cDNA量増やして半定量やればOKなんだなあ.
という学び.

このバンドをゲル切りしてシーケンス解析に出す!
